# Efficacy and safety of rechallenge with [^177^Lu]Lu-PSMA-I&T radioligand therapy in metastatic castration resistant prostate cancer

**DOI:** 10.1007/s00259-024-06905-5

**Published:** 2024-09-03

**Authors:** Giulia Santo, Gianpaolo Di Santo, Anna Sviridenko, Steffen Bayerschmidt, Lukas Wirth, Fabian Scherbauer, Peter Lehmann, Elisabeth von Guggenberg, Clemens Decristoforo, Isabel Heidegger-Pircher, Jasmin Bektic, Irene Virgolini

**Affiliations:** 1 Department of Nuclear Medicine, Medical University of Innsbruck, Anichstraße 35, Innsbruck, 6020 Austria; 2 Department of Experimental and Clinical Medicine, “Magna Graecia” University of Catanzaro, Catanzaro, Italy; 3 Department of Urology, Medical University of Innsbruck, Innsbruck, Austria

**Keywords:** [^177^Lu]Lu-PSMA-I&T, Prostate, mCRPC, RLT, Rechallenge

## Abstract

**Background:**

The purpose of this study was to evaluate the safety and outcome of rechallenge [^177^Lu]Lu-PSMA-I&T in newly progressed mCRPC patients after response to initial [^177^Lu]Lu-PSMA radioligand therapy (PRLT).

**Methods:**

We retrospectively included 18 patients who underwent rechallenge with [^177^Lu]Lu-PSMA-I&T. All patients presented with (i) newly progressed disease after response to initial PRLT; (ii) a [^68^Ga]Ga-PSMA-11 PET/CT confirming the presence of PSMA-positive metastases; iii) ECOG performance status 0–1. Adverse events were graded according to CTCAE v5.0. Response was assessed by PSA and classified according to PCWG3 recommendations. For patients who underwent restaging with [^68^Ga]Ga-PSMA-11 PET/CT, imaging response was categorised according to adapted PERCIST v1.0. In patients with discordant [^68^Ga]Ga-PSMA-11 PET/CT and PSA, other available imaging modalities were evaluated to confirm disease status. Overall survival (OS) was calculated from the first cycle of initial PRLT and rechallenge PRLT, respectively, until last patient contact or death.

**Results:**

Patients were initially treated with a median of 5 cycles (range 4–7) and were rechallenged after a median of 9 months (range 3–13). Each patient received a median of 4 (range 2–7) rechallenge cycles (median cumulative activity 26.1 GBq). None of the patients experienced life-threatening G4 adverse events during either treatment period. Grade 3 adverse events included one case of anaemia, one case of thrombocytopenia, and one case of renal failure. In 8/18 patients long-term toxicities were evaluated. Serious toxicities (≥ Grade 3) occurred in 3/8 patients (*n* = 1 G4 thrombocytopenia, *n* = 1 G4 renal failure and *n* = 1 pancytopenia and G4 renal failure). Best PSA50%-response was observed in 44% of patients and PSA-disease control was confirmed in 56% of patients at the last cycle. Of the 12/18 patients restaged by imaging, 6/12 (50%) patients had disease control (partial response/stable disease), 1/12 had a mixed response, and 5/12 had progression. After a median follow-up time of 25 months (range 14–44), 10 patients had died, 7 were still alive, and one patient was lost at follow-up. The median OS was 29 months (95%CI, 14.3–43.7 months) for the initial treatment and 11 months (95%CI, 8.1–13.8 months) for the first rechallenge course.

**Conclusion:**

More than half of patients benefit from rechallenge PRLT. Our analysis suggests that rechallenge may prolong survival in selected patients, with an acceptable safety profile.

**Supplementary Information:**

The online version contains supplementary material available at 10.1007/s00259-024-06905-5.

## Introduction

Prostate cancer (PCa) is the second most common cancer in men, with an estimated 1.4 million diagnoses and 397,000 deaths worldwide in 2022. In Europe, it is the most commonly diagnosed cancer and the third leading cancer-related cause of death [1].

Despite the availability of several treatments that can delay disease progression and prolong survival, advanced PCa presents with a poor prognosis, especially when considering patients with metastatic castration-resistant prostate cancer (mCRPC). In this setting, the efficacy of treatment decreases progressively, along with patient survival (i.e., overall survival (OS) of pretreated mCRPC patients ~ 11 months) [2].

Lutetium-177 [^177^Lu] prostate-specific membrane antigen (PSMA) radioligand therapy (PRLT) has shown encouraging results in mCRPC patients, prolonging both progression-free survival (PFS) and OS in the major controlled trials [3, 4], leading to the final approval of PRLT as the third-line systemic treatment for mCRPC [5].

In clinical practice, PSMA-617 and PSMA-I&T, two urea-based small molecule inhibitors of PSMA labelled with Lutetium-177, are commonly used for PRLT. Despite the differences in their chemical structure and the chelators used [6, 7], dosimetry data reported comparable mean tumour absorbed doses for both compounds [8], with no significant differences in terms of therapeutic efficacy and toxicity profile in clinical studies [9]. In addition, a recent systematic review and meta-analysis on dosimetry [10], revealed neither significant difference in tumour nor kidney absorbed dose between the two radiopharmaceuticals.

Generally, only mild side effects have been reported in patients undergoing PRLT, with the kidneys and bone marrow being the organs at risk [11]. In an investigator-initiated, single-institution phase 2 trial, for PRLT with [^177^Lu]Lu-PSMA-617 grade 3–4 lymphopenia, thrombocytopenia, anaemia, and neutropenia were reported in up to 32%, whereas for the kidneys, only grade 1–2 events occurred in 10% of patients [12]. Accordingly, Heck et al. showed in 100 patients treated with [^177^Lu]Lu-PSMA-I&T that treatment-related toxicities were mainly haematologic, including grade 3–4 toxicities, such as anaemia (9%), thrombocytopenia (4%) and neutropenia (6%), with no serious (≥ grade 3) non-haematologic toxicities such as renal failure [13]. Moreover, despite the high tracer accumulation in the salivary glands and consequently a higher absorbed dose compared to other normal organs [8], clinical experience showed only mild xerostomia with a variable rate (4 − 87% of patients) [14].

Nowadays, the approved treatment regimen for [^177^Lu]Lu-PSMA-617 is based on a fixed activity of 7.4 GBq, injected 5 to 7 weeks apart for up to 6 cycles, as a similar schedule can be applied for treatment with [^177^Lu]Lu-PSMA-I&T [15]. Dosimetry data indicate that an average of 40 GBq of [^177^Lu]Lu-PSMA-I&T is safe and justifiable [16], considering 23 Gy as the dose limit to the kidney [17, 18]. Moreover, our previous experience with [^177^Lu]Lu-PSMA-617 [19] demonstrated an average absorbed dose of 0.77 ± 0.53 Gy to the bone marrow, suggesting that the tolerable administered activity should lie around 45 GBq without exceeding the generally recommended threshold of 2 Gy. To note, these absorbed dose limits applied to radioligand therapy are based on external-beam radiation therapy (EBRT), which is unlikely to predict the toxicities of systemic radionuclide treatment. Based on experience with neuroendocrine tumours [20], patients without risk factors for kidney disease, might tolerate a renal biologic equivalent dose (BED) up to 40 Gy.

In addition, the PRLT schedule, which was largely adapted from previous experience with peptide receptor radionuclide therapy (PRRT) for neuroendocrine tumours, does not take into account the different nature of the prostate cancer disease and the therapeutic options available at this advanced stage. Namely, most patients who respond to the first course of PRLT and relapse even after a short period of time have no further treatment options in most of the cases. Therefore, rechallenge with PRLT should be considered in this patient population. Based on general oncological treatment principles, rechallenge refers to “a patient who relapses (has progressive disease after an initial response to therapy) after discontinuation of therapy and is re-treated with the same therapy - typically with the same dose and regimen - after a treatment-free interval” [21].

Data on the efficacy of rechallenge with PRLT are scarce but promising, showing a good response rate in previously treated patients, with PSA50% responses ranging from 73 to 37.1% [22–25] and a cumulative OS from the first cycle of the initial period of up to 40 months [25].

An acceptable safety profile, almost comparable to the initial treatment, was also demonstrated [22–25], with anaemia and thrombocytopenia being the most commonly reported G3-G4 haematologic adverse events, and high-grade renal toxicity occurring in a limited number of patients (i.e., 7.4% [25]).

To note, most of the published data are based on the use of [^177^Lu]Lu-PSMA-617 and only limited clinical experience is reported on rechallenge with [^177^Lu]Lu-PSMA-I&T, probably due to the use of the approved radiopharmaceutical [^177^Lu]Lu-PSMA-617 (Pluvicto ^®^) in major clinical trials.

Therefore, the aim of this retrospective study was to evaluate response and survival outcome in newly progressive mCRPC patients who underwent rechallenge with [^177^Lu]Lu-PSMA-I&T after an initial response to PRLT. In addition, the safety profile was reported and described.

## Methods

### Patients

We retrospectively included 18 patients who underwent rechallenge [^177^Lu]Lu-PSMA-I&T at the Department of Nuclear Medicine at the Medical University of Innsbruck. All patients analysed presented with (i) new progression of mCRPC after response to initial PRLT; (ii) no history of grade 4 adverse events during the first period of PRLT; (iii) a [^68^Ga]Ga-PSMA-11 PET/CT before the start of rechallenge confirming the presence of PSMA-positive metastases; (iv) Eastern Cooperative Oncology Group (ECOG) performance status 0–1. Assessment of patient-reported well-being (i.e., fatigue, pain, level of activity) was considered a key parameter for restarting the therapy or not. Patient data, medical history (i.e., previous treatments and current medication for prostate cancer, comorbidities), and general clinical condition at the time and during treatment were retrieved from our clinical record.

All procedures performed in this study were in accordance with the principles of the 1964 Declaration of Helsinki and its subsequent amendments [26]. [^177^Lu]Lu-PSMA-I&T was prepared according to the Austrian Medicinal Products Act AMG § 8 and § 62 [27] and all regulations of the Austrian Agency for Radiation Protection were observed [28]. The study evaluation followed an intention-to-treat approach in all patients, and patients were followed up until death.

### Radiopharmaceutical preparation

PSMA I&T was obtained from piCHEM (Raaba-Grambach, Austria). [^177^Lu]LuCl3 was obtained in n.c.a. quality (EndolucinBeta^®^, ITM Medical Isotopes GmbH, Garching, Germany). [^177^Lu]Lu-PSMA I&T was prepared as previously described [29]. The final product met the specifications of the European Pharmacopoeia [30].

### Treatment administration

Sufficient hydration was ensured by intravenous infusion (i.e. 1000 ml of 0.9% saline at a rate of 300 ml/h) starting 30 min in advance and 2 h following administration. In compliant patients, oral hydration was also suggested. Prophylactic antiemetic therapy (i.e. ondansetron 20mgx2) 20 min before treatment administration and 3–4 h after treatment was administered. No specific measures to minimise xerostomia were adopted. [^177^Lu]-PSMA-I&T was administered intravenously (flow 100 ml/h, 100 ml) over 1 h by a dedicated peristaltic infusion pump system.

After treatment patients were invited to void frequently during the first 6–10 h in order to reduce bladder dose. According to the Austrian Radiation Protection laws, all patients were treated as in-patients at the Nuclear Medicine ward and could be discharged at 48 h post-injection. As suggested by the European Association of Nuclear Medicine (EANM) guideline [16], a planar post-therapeutic emission scan was obtained at 24 h after injection to rule out extravasation and to confirm physiological tracer biodistribution/excretion.

### Toxicity assessment

Clinical examinations were done prior to each cycle and before discharge. Laboratory tests were carried out before every treatment infusion and at least until two months after the last infusion at every follow-up visit. Lab examination included: blood cell count, creatinine, eGFR, AST/GOT, ALT/GPT, total bilirubin, albumin, ALP, LDH, PSA, and CRP. Adverse events were graded according to the Common Terminology Criteria for Adverse Events (CTCAE v5.0) [31]. Clinical report included vital parameters and other possible side effects (such as xerostomia, nausea, vomiting, pain, tiredness, fatigue) using the standard hospital monitoring and documentation systems during their residence. Response criteria of the Eastern Cooperative Oncology Group (ECOG) performance status were used to assess quality of life (QoL). The ECOG status ranged from 0 to 1 (0 = fully active and 5 = dead). All clinical and biochemical data were retrospectively retrieved from our electronic medical records.

### Response assessment

The biochemical response was classified according to the recommendations of the Prostate Cancer Working Group 3 (PCWG3) as follows: partial response (PR) if there was a PSA-decrease ≥ 50% and progressive disease (PD) if PSA increased ≥ 25%. As stable disease (SD) was regarded as either PSA-increase < 25% or PSA-decrease < 50% [32]. Beyond the overall (best) PSA response after the start of treatment, PSA50%-response was evaluated also considering the PSA level at the last cycle of treatment and two months after the last PRLT cycle.

Imaging assessment was performed by [^68^Ga]Ga-PSMA-11 PET/CT two months after the last cycle of rechallenge PRLT. The imaging response was classified according to the adapted PERCIST 1.0 criteria [33, 34]. In addition, mixed response (MxR) was considered as a partial/complete response of the known metastases and, at the same time, the occurrence of new lesions. For the MxR, the overall tumour load should be either stable or less in comparison to the pre-therapeutic imaging. In patients presented with discordant [^68^Ga]Ga-PSMA-11 PET/CT and PSA findings, other available imaging modalities (i.e., contrast-enhanced CT, [^18^F]FDG PET/CT, [^68^Ga]Ga-DOTATOC] were evaluated to confirm disease status. Disease control rate (DCR) was defined as the sum of CR/PR and SD.

### Statistical analysis

Statistical analysis was performed with IBM SPSS Statistics, version 26 (IBM Corp., Armonk, N.Y., USA) and GraphPad Prism version 10.0.0 (GraphPad Software, Boston, Massachusetts USA). Microsoft Excel (Microsoft Office 2010) was used for water-flow analyses of the PSA changes. Categorical and continuous variables were analysed using descriptive statistics. The paired sample t-test was used to compare interindividual changes in continuous biochemical parameters. OS was estimated with the Kaplan-Meier method (censored data) and calculated from the first cycle of initial PRLT and rechallenge PRLT, respectively, until last patient contact or death.

## Results

### Patients and treatment

A total of 18 patients were treated with a second course of [^177^Lu]Lu-PSMA-I&T between March 2020 and December 2023. Prior to the rechallenge, most of the patients (89%, *n* = 16) had lymph node metastases on [^68^Ga]Ga-PSMA-11 PET/CT. In addition, the majority of patients had previously received chemotherapy (67%, *n* = 12) and the remaining *n* = 6 patients were chemo-naïve. All patients were on androgen deprivation therapy (ADT) at the time of rechallenge. Regular use of pain medication was reported by 33% (*n* = 6) of patients.

Patients were initially treated with a median of 5 cycles (range 4–7; median cumulative activity of the first period: 38.4 GBq) and were rechallenged after a median of 9 months (range 3–13). Each patient received a median of 4 (range 2–7) rechallenge cycles (median cumulative activity of the second period: 26.1 GBq). Baseline characteristics of the patients are summarised in Table 1.

**Table 1:** Patients characteristics before re-challenge PRLT (*n* = 18 patients)

Characteristic	
Age (y)	70 (44–93)
Gleason Score	9 (7–10)
N° cycles first course PRLT	5 (4–6)
Cumulated administered activity first course PRLT (GBq)	38.4 ± 8.76
PSA (ng/ml)	186 ± 528
Alkaline phosphatase (U/L)	169.8 ± 168.4
Haemoglobin (g/L)	112 ± 18
Lactate dehydrogenase (U/L)	275.5 ± 174.0
Leucocyte (g/L)	5.7 ± 1.8
Platelet (g/L)	219.9 ± 62.6
Prior therapies	
Docetaxel	11 (61%)
Cabazitaxel	5 (28%)
Abiraterone and/or Enzalutamide	11 (61%)
[^223^Ra]RaCl_2_	3 (17%)
Pain medication	
Regular intake	6 (33%)
Stage of disease [^68^Ga]Ga-PSMA-11	
Node	16 (89%)
Bone	13 (72%)
< 6 lesions	2 (15%)
6–20 lesions	3 (23%)
> 20 lesions	5 (38%)
diffuse/super scan	3 (23%)
Visceral	4 (22%)
Liver	1 (25%)
Lung	2 (50%)
Brain	1 (25%)

### Response and survival

**Fig. 1:**
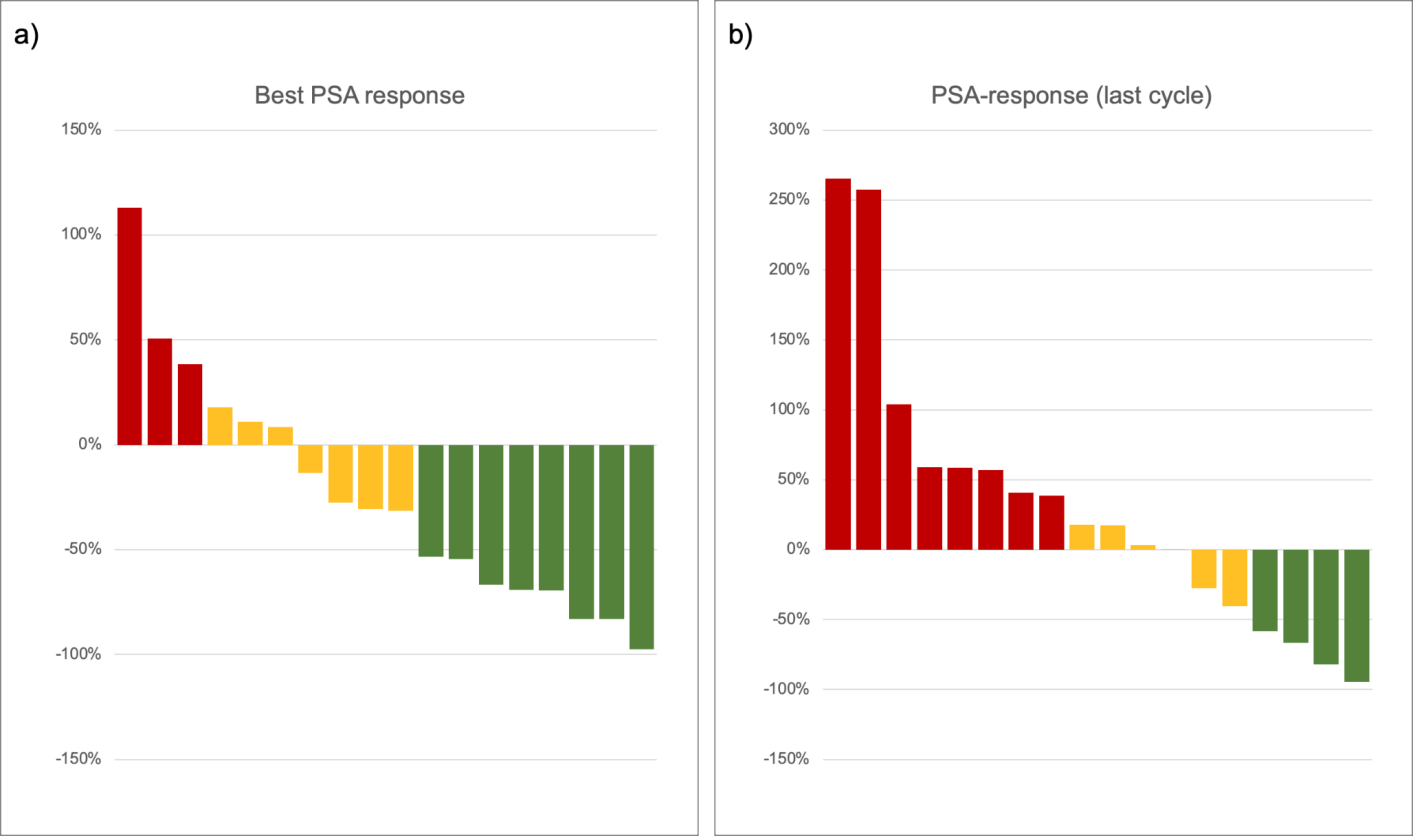
Waterfall plots of the best PSA response after the start of rechallenge (**a**) and the corresponding value at the last cycle measurement (**b**)

Considering the best overall PSA50% response after the start of rechallenge treatment, PR was observed in 8/18 (44%) of patients, SD in 7/18 (39%) of patients, and PD in 3/18 (17%) patients. While, at the last cycle of treatment, a PSA50% response was observed in 4/18 (22%) patients, 6/18 (34%) patients were considered as SD according to their PSA decrease at rechallenge (− 40% < PSA range < + 18%) and the remaining 8/18 (44%) patients showed a PSA increase above 25%.The DCR at the last cycle measurement was 56%. Figure 1 shows the waterfall plots of the best PSA response after the first rechallenge cycle and the corresponding value considering the last treatment administration, respectively.

At two months, PSA levels were available in 14/18 patients. Of these, 3/14 (21%) were PR, 5/14 (36%) SD, and 6/14 (43%) PD, confirming a DCR in more than half of the patients. Of the remaining 4 patients, 3 died prior to restaging and 1 patient was lost at follow-up.

**Fig. 2:**
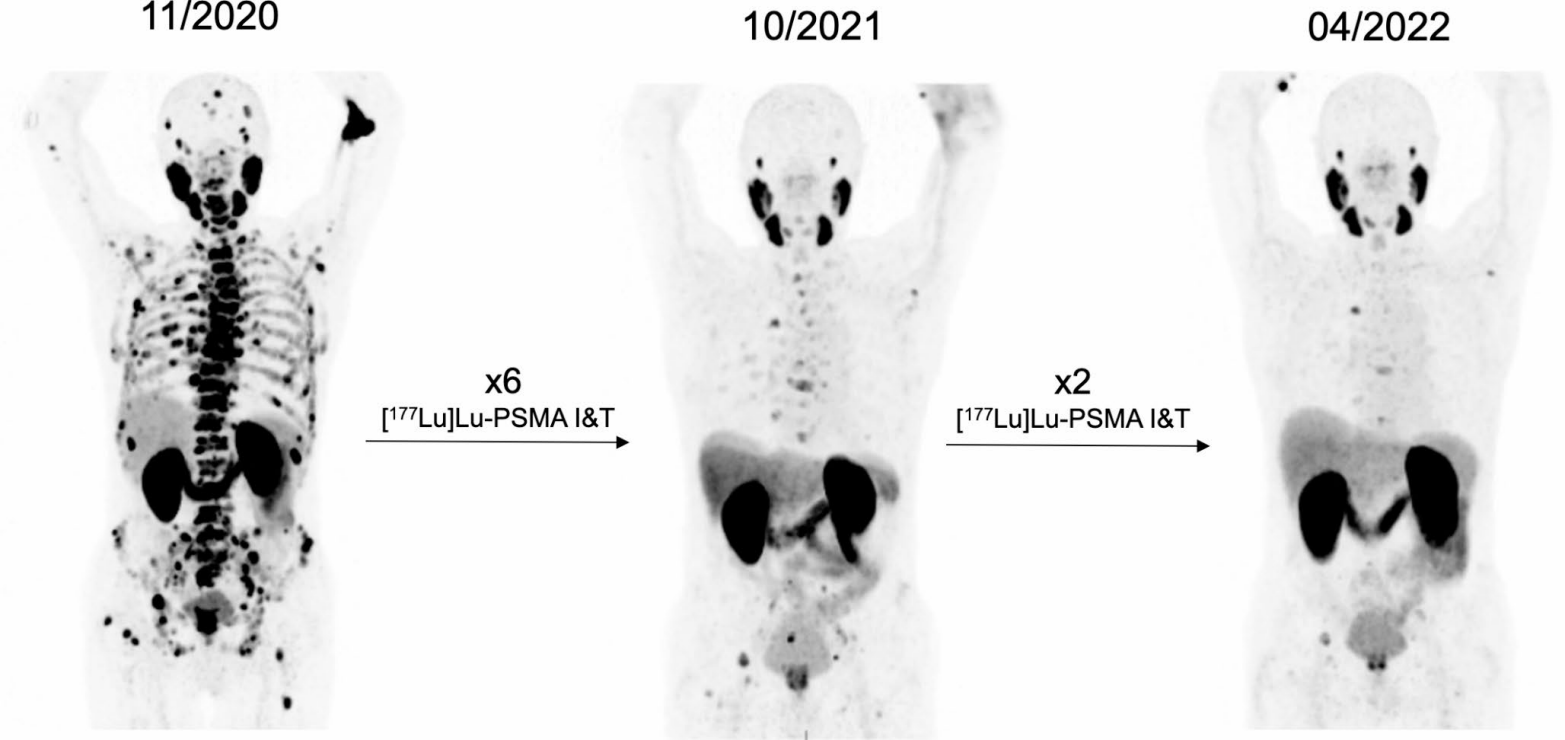
Case example of a patient who responded to the first treatment course of therapy and continues to benefit from rechallenge PRLT. A PSA decline of ~ 83% was registered at the end of the second course of therapy

**Fig. 3:**
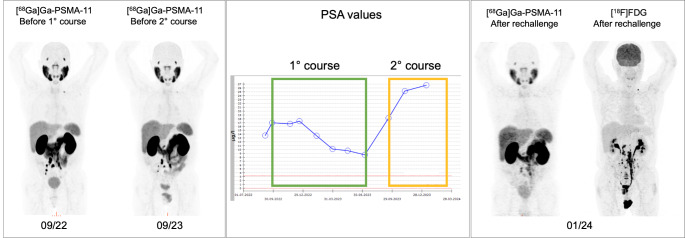
Case example of a patient with progressive disease at the second course of PRLT. The restaging [^68^Ga]Ga-PSMA-11 PET/CT showed a partial response with the disappearance of some lesions in contrast to the increase of PSA values over time. Thus, the progression of the disease was confirmed by [^18^F]FDG PET/CT

Of the 12/18 patients restaged by imaging, 6/12 (50%) patients showed disease control (3 PR, 3 SD), 1/12 MxR, and 3/12 PD on [^68^Ga]Ga-PSMA-11 PET/CT consistent with increasing PSA levels. In the remaining two patients, PD was confirmed by other available imaging modalities. Thus, PD was confirmed by cross-sectional imaging in 5/12 (42%) patients. Two case examples are shown in Figs. 2 and 3.

**Fig. 4:**
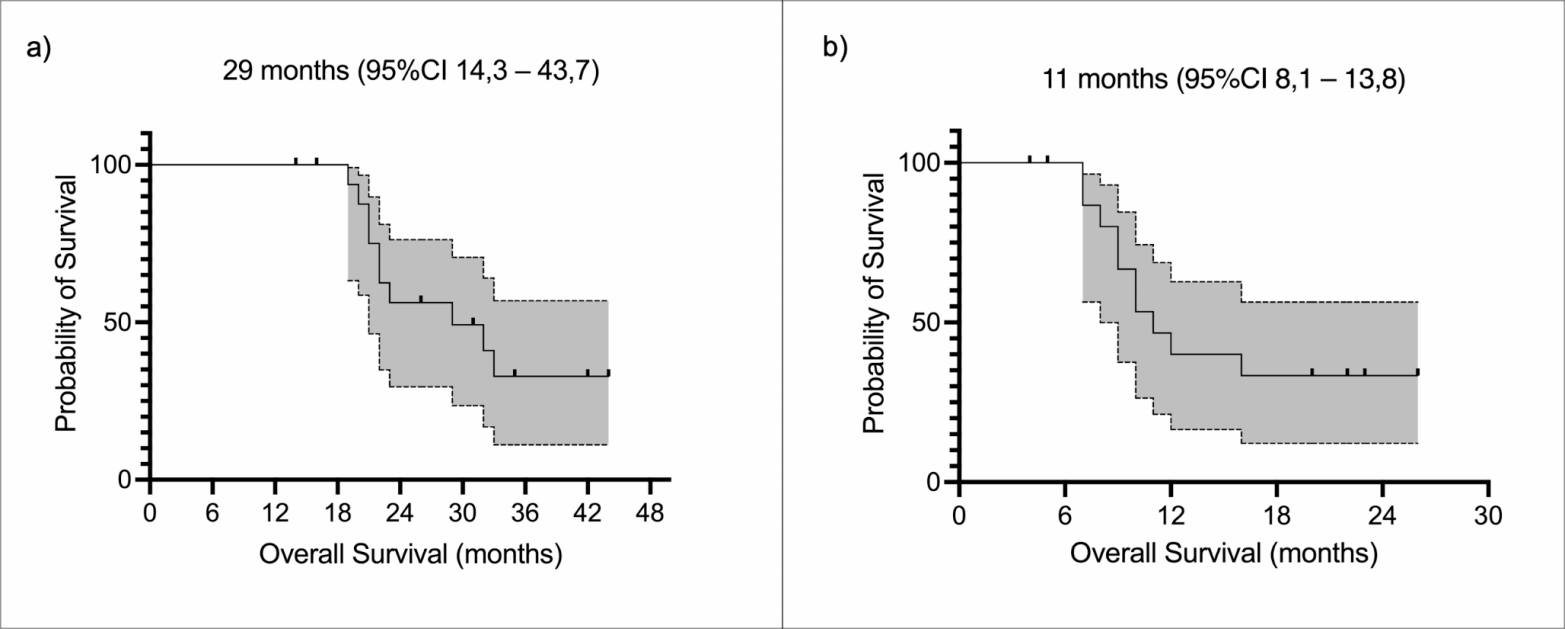
Kaplan-Meier curves of overall survival from the first treatment cycle (**a**) and from rechallenge (**b**)

After a median follow-up time of 25 months (range 14–44), 10 patients had died, 7 were still alive, and 1 was lost at follow-up. The median OS was 29 months (95%CI, 14.3–43.7 months) for the first treatment and 11 months (95%CI, 8.1–13.8 months) for the first rechallenge course. The Kaplan-Meier survival curves are shown in Fig. 4.

In addition, no statistically significant difference in OS was observed between patients who received chemotherapy prior to rechallenge and chemo-naïve patients, whether considering cumulative OS from the start of initial treatment (median OS 29 vs. 33.5 months, *p* = 0.62) or from the start of rechallenge (median OS 11 vs. 17.5 months, *p* = 0.61). Kaplan-Meier curves are shown in Supplementary file 1.

### Safety profile

**Fig. 5:**
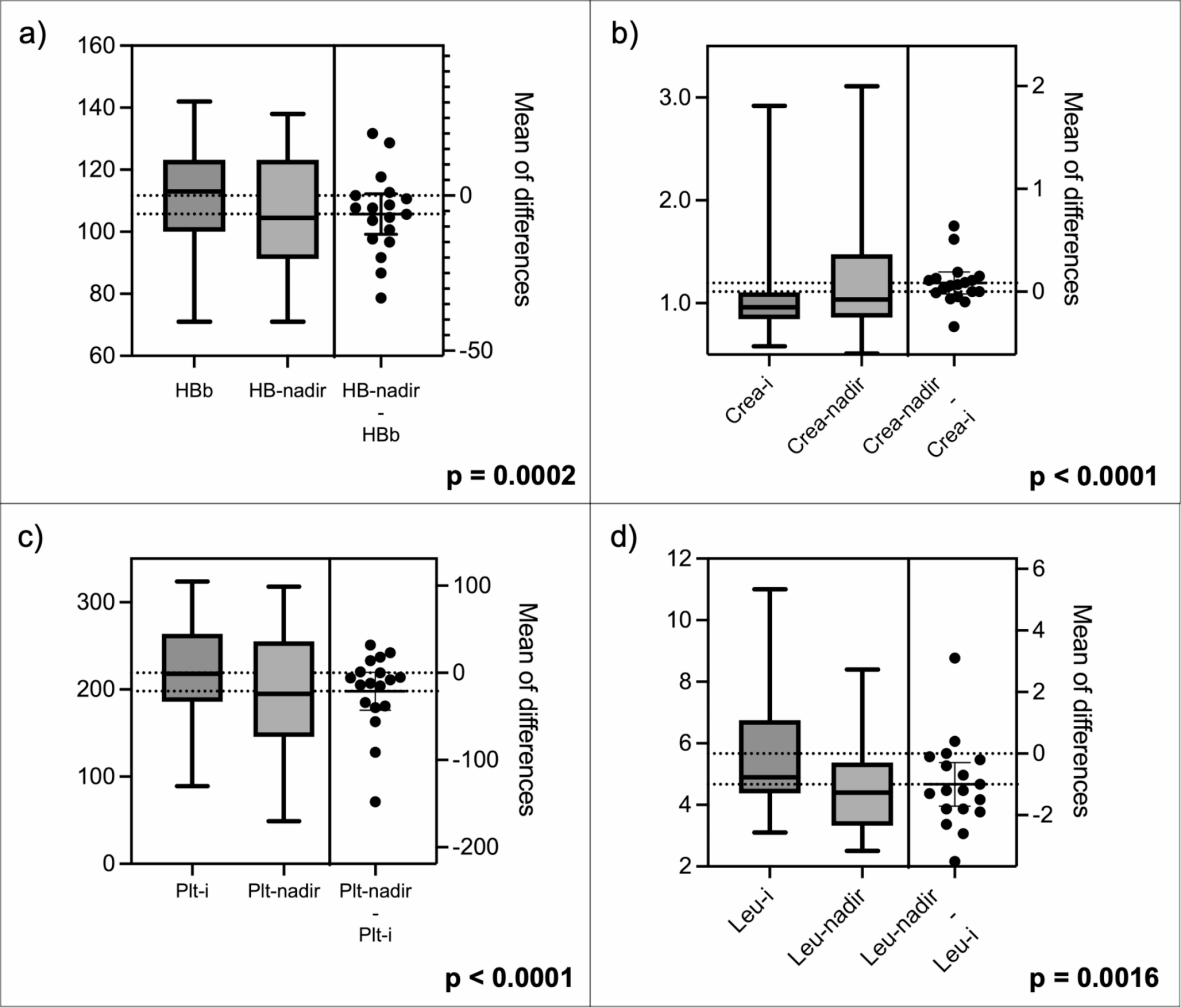
Box Plots show comparison of the interindividual changes in main biochemical parameters considered for toxicities

The inter-individual changes of the main biochemical parameters are shown in Fig. 5. Haematological parameters showed a significant decrease over the course of rechallenge PRLT. None of the patients experienced life-threatening G4 adverse events during either treatment period. Grade 3 adverse events included one case of anaemia, one case of thrombocytopenia, and one case of renal failure (3/18 patients, 16%). Anaemia of any grade was the most common adverse event, occurring in 9/18 (50%) patients. Only mild xerostomia, nausea/vomiting or anorexia were reported during treatment.

Long-term toxicities were assessed in 8/18 (44%) patients. During a median follow-up time of 208 days (range 72–620 days) after the last rechallenge cycle, serious toxicities (≥ grade 3) occurred in 3/8 patients (*n* = 1 G4 thrombocytopenia, *n* = 1 G4 renal failure and *n* = 1 patient with pancytopenia and G4 renal failure). In addition, *n* = 5 patients experienced G3 anaemia.

Considering the two patients with G4 renal toxicity, in the first case G3 renal toxicity was already present during treatment and progressed to G4 9 months after the last treatment cycle. However, the patient with progressive disease died one day after the renal toxicity worsened.

**Fig. 6:**
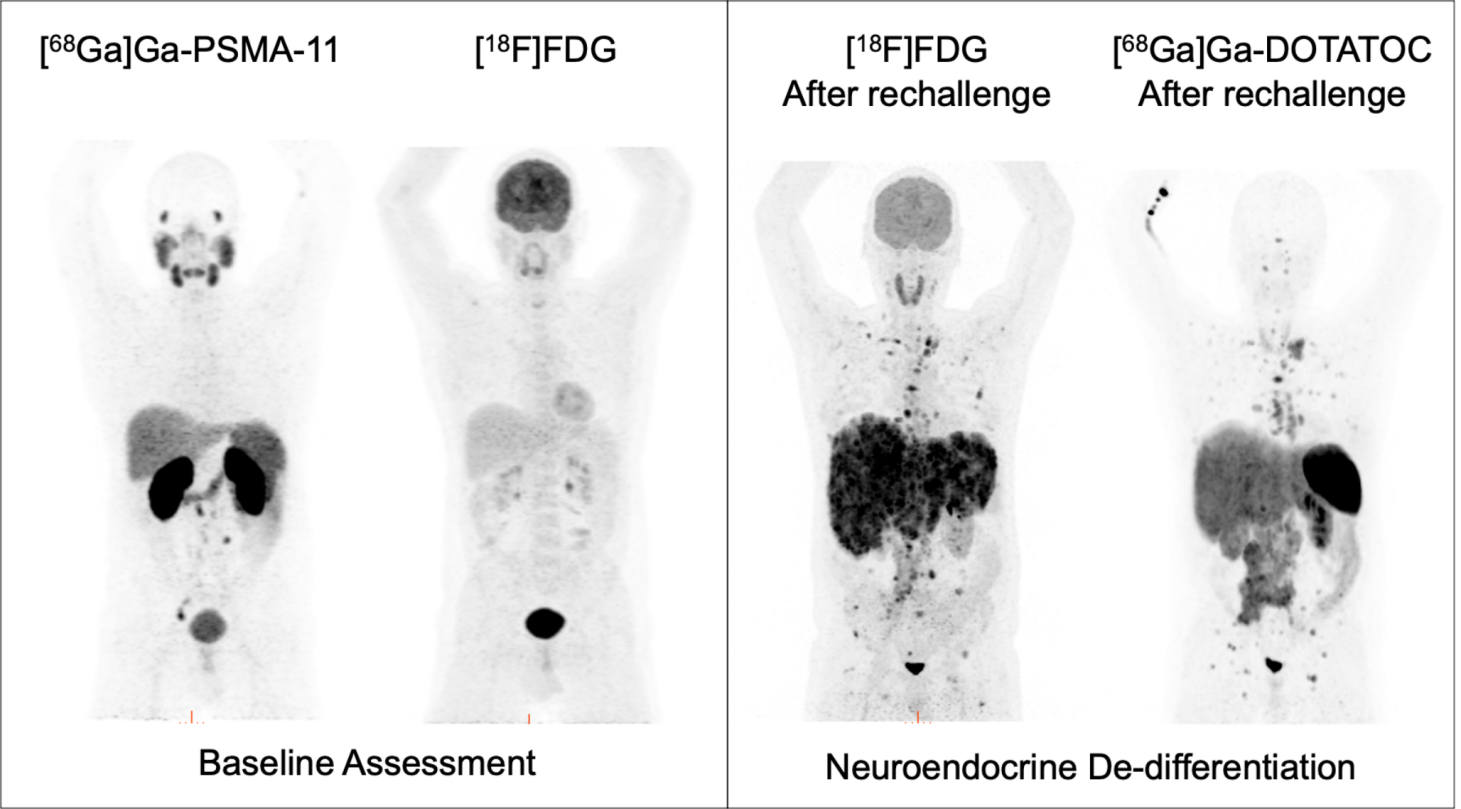
A case example of neuroendocrine differentiation after PRLT. The patient with prostate adenocarcinoma GS 9 (5 + 4), was diagnosed in 2020. The patient underwent baseline assessment with both [^68^Ga]Ga-PSMA and [^18^F]FDG PET/CT (left panel) prior to [^177^Lu]Lu-PSMA-I&T therapy (6 cycles, April-December 2022) with a PSA reduction of approximately 50%. After 2 months, new lymph node progression was assessed by [^68^Ga]Ga-PSMA PET/CT (no [^18^F]FDG PET/CT performed before rechallenge). Thus, the patient underwent rechallenge 5 months after the last cycle. After the second rechallenge cycle, the patient showed progressive clinical deterioration despite low PSA levels. Therefore, treatment was discontinued and the patient was re-evaluated with [^18^F]FDG and [^68^Ga]Ga-DOTATOC PET/CT (right panel), which showed neuroendocrine de-differentiation of the disease

In the second case, G4 renal toxicity occurred 7 months after the end of treatment together with pancytopenia, and the patient died 23 days after the haematologic laboratory findings and 2 days after the onset of G4 renal toxicity. The patient’s case is illustrated in Fig. 6, as an example of progressive de-differentiated disease.

High-grade adverse events according to CTCAE version 5.0 are shown in Table 2.

**Table 2:** High-grade toxicities during rechallenge PRLT (*n* = 18 patients) and after treatment (*n* = 8 patients) based on CTCAE version 5

Parameters	During rechallenge (*n* = 18)		After rechallenge (*n* = 8)*	
	G3	G4	G3	G4
Haemoglobin (g/L)	1	0	5	0
Platelet (g/L)	1	0	0	2
Leukocyte (g/L)	0	0	1	1
Creatinine (mg/dl)	1	0	0	2

* pancytopenia (G3 anaemia, G4 thrombocytopenia, G4 leukopenia) and a case of G4 renal failure occurred in the same patient

## Discussion

The relatively high number of patients who continue to benefit from PRLT, together with the low rate of severe toxicities, reflects the potentially promising role of a second course of PRLT in mCRPC patients. In our analysis based on 18 patients rechallenged with [^177^Lu]Lu-PSMA-I&T, we showed that more than half of the patients continued to benefit from PRLT. In terms of PSA response, other authors have reported similar results. The Australian study [23] in a subset of 15 patients rechallenged with [^177^Lu]Lu-PSMA-617, showed that 73% of patients had a PSA decrease of at least 50%. The authors considered the best PSA response as the endpoint. In our analysis, the best PSA response from the baseline was seen in 44% of patients. However, the number of patients with a PSA50% response at the last cycle measurement decreased to 22%. A similar consideration can be found in the paper by Yordanova and colleagues [24] on 30 patients rechallenged with [^177^Lu]Lu-PSMA-617. In their study, the evaluation of PSA decline over cycles showed how the number of patients maintaining a PSA50% response decreased progressively with the number of cycles: a PSA decline ≥ 50% was seen in 26, 40, and 20% of the first, second and third rechallenge cycles, respectively.

PSA levels alone are known to be of limited value in assessing treatment response in mCRPC patients [35]. However, PSA changes are often associated with response to PRLT [36] and serve as a surrogate for response during treatment [37]. Although an overall positive relationship between higher PSA decline rates and outcome is likely, data on the optimal threshold for PSA decline remain sparse [38], mainly considering that the recommendation used to assess PSA response (i.e., PCWG3) is mostly derived from patients undergoing chemotherapy with taxane. Indeed, although the majority of the studies adopted the recommended threshold of 50%, some other authors argued that lower thresholds (e.g., 30%) should be more accurate to assess the response to PRLT [36, 39].

In our population, the disease control rate based on PSA decline was confirmed in 56% of patients, even taking into account the PSA value at the time of the last treatment cycle, reflecting an overall benefit of the rechallenge in more than half of the patients.

Based on these considerations and the setting of the patients analysed, on the one hand we should evaluate different thresholds for assessing PSA response. On the other hand, we should also consider that some patients may become resistant to treatment over time and a single time point measurement may not reflect the changes in PSMA expression during treatment. This point also opens the way for further considerations regarding the assessment of response to PRLT by imaging.

Specifically, our study emphasised the need for cross-sectional imaging to assess response in mCRPC patients under PRLT and the relative difficulty of using [^68^Ga]Ga-PSMA-11 PET/CT alone. Namely, the exclusive use of [^68^Ga]Ga-PSMA-11 PET/CT for response assessment may limit the evaluation of disease and underestimate the progressive dedifferentiation that can often occur at this stage.

This led to the consideration that most of the existing response criteria for PRLT (such as aPERCIST 1.1, RECIP 1.0, PPP) may be limited by their assumption of a persistent PSMA expression throughout the course of the disease. Indeed, neuroendocrine dedifferentiation and progression with a loss of PSMA expression are not defined, and progression without a finding on PSMA PET or CT is not included in such criteria [40]. In fact, as shown in our study, only the use of complementary images allowed to correctly define the disease status (Figs. 3 and 6). This was clearly demonstrated in one of our rechallenged patients (Fig. 6), where the use of complementary imaging modalities ([^18^F]FDG PET/CT and [^68^Ga]Ga-DOTATOC PET/CT) showed disease progression, overcoming the limitations associated with PSA measurements and [^68^Ga]Ga-PSMA-11 PET/CT alone. Therefore, caution should be observed in this group of patients and the evaluation of response should always include the use of additional diagnostic tools, as suggested by the EANM guidelines [16, 41, 42]. In this context, a complete clinical and laboratory evaluation should always guide the clinician.

In PCa, neuroendocrine differentiation (NED) is recognised as an adaptive mechanism that allows PCa cell populations to escape treatment [43, 44]. Increasing evidence suggests that in addition to ADTs [45], treatment-related neuroendocrine PCa (t-NEPC) can also be induced by radio- [46, 47] and chemotherapy [47]. In the aforementioned patient, extracted from our population, it is difficult to state clearly that PRLT induced neuroendocrine transformation because of the lack of neuroendocrine biomarkers assessment before the start of rechallenge. However, a thorough clinical experience with accurate imaging and biochemical evaluation before and during treatment is warranted to describe the impact of t-NEPC in patients undergoing PRLT.

Another confirmatory finding of our study is the OS of these patients. We reported a median OS from the first treatment cycle of 29 months (95%CI, 14.3–43.7 months), which is consistent with other published results on the rechallenge cohort [23–25]. When compared to the survival results of the VISION trial [3], a real benefit in cumulative OS was observed. Certainly, the rechallenge results reported in our study and others were partly influenced by the characteristics of the sample, which included patients who had previously responded to PRLT. However, the possibility of prolonging treatment [25, 48] or rechallenging patients who benefit from the first course appears to be reliable, especially in those patients who have no alternative treatment options.

This finding is also supported by the low rate of toxicities reported. Indeed, only 3 patients experienced G3 toxicities and no treatment-related deaths were recorded according to the retrospective revision of our data. It’s important to note that in patients followed-up after the end of treatment, all high-grade toxicities occurred in patients with late-stage progressive disease and appeared to be related to disease status rather than treatment. In this setting, the toxicity profile is consistent with other reports. Namely, a recent multicenter German retrospective study reported one case of G4 thrombocytopenia and confirmed G3 anaemia as the most frequent haematological toxicity [25]. Regarding renal toxicities, the German group reported 5 cases of G3 decrease in GFR in the rechallenge subgroup. However, to the best of our knowledge, we are the first to report a long-term analysis of toxicities (median follow-up of 208 days after the end of treatment) in rechallenged patients. Two cases of G4 renal toxicity were reported in our study, but these findings were in patients with progressive disease and G3 renal toxicity that progressed to G4 only a few days before death. Our study has several limitations that have to be considered, above all its retrospective nature and the relatively small sample size. The prevalent lymph node involvement in our cohort could affect the results due to the better outcome associated with this subgroup of patients [49]. In addition, the lack of re-staging imaging in all patients could be a limitation in assessing response. However, many patients died due to disease progression before the time of re-staging PET/CT or were lost at follow-up. Finally, dosimetry data could represent an additional endpoint for future investigation to correctly identify the absorbed tumour dose and associated treatment toxicities, mainly considering the prolonged OS in patients who were rechallenged. Moreover, the introduction of targeted alpha therapy (TAT) such as [^225^Ac]Ac-PSMA-617 in mCRPC should also be considered in the future when a low biologically effective dose (BED) is achieved with Lutetium-177 [50].

## Conclusion

Our results suggest that rechallenge with [^177^Lu]Lu-PSMA-I&T can be a reliable option in patients who responded to the first course of PRLT, with half of them benefiting from the treatment. In addition, rechallenge demonstrated to prolong survival in selected patients with an acceptable safety profile. However, caution should be made with regard to PSA levels and imaging tools used to assess response at this stage of disease. Prospective studies are warranted to support these preliminary results and ultimately assess the utility of rechallenge with PRLT in patients who benefit from the first course of treatment.

## Electronic supplementary material

Below is the link to the electronic supplementary material.

Supplementary Material 1

## Data Availability

The datasets generated during and/or analyzed during the current study are available from the corresponding author upon reasonable request.

## References

[1] Global Cancer Observatory https:https://gco.iarc.fr/en (accessed on 20th April 2024).

[2] Hussain M, Goldman B, Tangen C, et al. Prostate-specific antigen progression predicts overall survival in patients with metastatic prostate cancer: data from Southwest Oncology Group trials 9346 (Intergroup Study 0162) and 9916. J Clin Oncol. 2009;27(15):2450–6. 10.1200/JCO.2008.19.9810. 19380444 10.1200/JCO.2008.19.9810 PMC2684851

[3] Sartor O, de Bono J, Chi KN, et al. Lutetium-177-PSMA-617 for metastatic castration-resistant prostate Cancer. N Engl J Med. 2021;385(12):1091–103. 10.1056/NEJMoa2107322. 34161051 10.1056/NEJMoa2107322 PMC8446332

[4] Hofman MS, Emmett L, Sandhu S, et al. [^177^Lu]Lu-PSMA-617 versus cabazitaxel in patients with metastatic castration-resistant prostate cancer (TheraP): a randomised, open-label, phase 2 trial. Lancet. 2021;397(10276):797–804. 10.1016/S0140-6736(21)00237-3. 33581798 10.1016/S0140-6736(21)00237-3

[5] Fallah J, Agrawal S, Gittleman H, et al. FDA approval Summary: Lutetium Lu 177 Vipivotide Tetraxetan for patients with metastatic castration-resistant prostate Cancer. Clin Cancer Res. 2023;29(9):1651–7. 10.1158/1078-0432.CCR-22-2875. 36469000 10.1158/1078-0432.CCR-22-2875 PMC10159870

[6] Benešová M, Schäfer M, Bauder-Wüst U, et al. Preclinical evaluation of a tailor-made DOTA-Conjugated PSMA inhibitor with optimized Linker Moiety for imaging and endoradiotherapy of prostate Cancer. J Nucl Med. 2015;56(6):914–20. 10.2967/jnumed.114.147413. 25883127 10.2967/jnumed.114.147413

[7] Weineisen M, Schottelius M, Simecek J, et al. 68Ga- and ^177^Lu-Labeled PSMA I&T: optimization of a PSMA-Targeted Theranostic Concept and First Proof-of-Concept Human studies. J Nucl Med. 2015;56(8):1169–76. 10.2967/jnumed.115.158550. 26089548 10.2967/jnumed.115.158550

[8] Schuchardt C, Zhang J, Kulkarni HR, Chen X, Müller D, Baum RP. Prostate-specific membrane Antigen Radioligand Therapy using ^177^Lu-PSMA I&T and ^177^Lu-PSMA-617 in patients with metastatic castration-resistant prostate Cancer: comparison of Safety, Biodistribution, and Dosimetry. J Nucl Med. 2022;63(8):1199–207. 10.2967/jnumed.121.262713. 34887335 10.2967/jnumed.121.262713 PMC9364353

[9] Hartrampf PE, Weinzierl FX, Buck AK, et al. Matched-pair analysis of [^177^Lu]Lu-PSMA I&T and [^177^Lu]Lu-PSMA-617 in patients with metastatic castration-resistant prostate cancer. Eur J Nucl Med Mol Imaging. 2022;49(9):3269–76. 10.1007/s00259-022-05744-6. 35243517 10.1007/s00259-022-05744-6 PMC9250457

[10] Ells Z, Grogan TR, Czernin J, Dahlbom M, Calais J. Dosimetry of [^177^Lu]Lu-PSMA-Targeted Radiopharmaceutical therapies in patients with prostate Cancer: a comparative systematic review and metaanalysis. J Nucl Med Published Online July. 2024;3. 10.2967/jnumed.124.267452. 10.2967/jnumed.124.267452 PMC11294071 38960712

[11] Sadaghiani MS, Sheikhbahaei S, Werner RA, et al. A systematic review and Meta-analysis of the effectiveness and toxicities of Lutetium-177-labeled prostate-specific membrane Antigen-targeted Radioligand Therapy in Metastatic Castration-resistant prostate Cancer. Eur Urol. 2021;80(1):82–94. 10.1016/j.eururo.2021.03.004. 33840558 10.1016/j.eururo.2021.03.004 PMC8206006

[12] Hofman MS, Violet J, Hicks RJ, et al. [^177^Lu]-PSMA-617 radionuclide treatment in patients with metastatic castration-resistant prostate cancer (LuPSMA trial): a single-centre, single-arm, phase 2 study. Lancet Oncol. 2018;19(6):825–33. 10.1016/S1470-2045(18)30198-0. 29752180 10.1016/S1470-2045(18)30198-0

[13] Heck MM, Tauber R, Schwaiger S, et al. Treatment outcome, toxicity, and predictive factors for Radioligand Therapy with ^177^Lu-PSMA-I&T in metastatic castration-resistant prostate Cancer. Eur Urol. 2019;75(6):920–6. 10.1016/j.eururo.2018.11.016. 30473431 10.1016/j.eururo.2018.11.016

[14] Fuoco V, Argiroffi G, Mazzaglia S, et al. Update on radioligand therapy with ^177^Lu-PSMA for metastatic castration-resistant prostate cancer: clinical aspects and survival effects. Tumori. 2022;108(4):315–25. 10.1177/03008916211037732. 34405748 10.1177/03008916211037732

[15] Kratochwil C, Fendler WP, Eiber M, et al. Joint EANM/SNMMI procedure guideline for the use of ^177^Lu-labeled PSMA-targeted radioligand-therapy (^177^Lu-PSMA-RLT). Eur J Nucl Med Mol Imaging. 2023;50(9):2830–45. 10.1007/s00259-023-06255-8. 37246997 10.1007/s00259-023-06255-8 PMC10317889

[16] Okamoto S, Thieme A, Allmann J, et al. Radiation Dosimetry for ^177^Lu-PSMA I&T in metastatic castration-resistant prostate Cancer: absorbed dose in normal organs and Tumor lesions. J Nucl Med. 2017;58(3):445–50. 10.2967/jnumed.116.178483. 27660138 10.2967/jnumed.116.178483

[17] Maffey-Steffan J, Scarpa L, Svirydenka A et al. The ^68^Ga/^177^Lu-theragnostic concept in PSMA-targeting of metastatic castration-resistant prostate cancer: impact of post-therapeutic whole-body scintigraphy in the follow-up [published correction appears in Eur J Nucl Med Mol Imaging. 2020;47(3):740. doi: 10.1007/s00259-019-04660-6]. *Eur J Nucl Med Mol Imaging*. 2020;47(3):695–712. 10.1007/s00259-019-04583-2. 10.1007/s00259-019-04583-2 PMC7005064 31776632

[18] Emami B, Lyman J, Brown A, et al. Tolerance of normal tissue to therapeutic irradiation. Int J Radiat Oncol Biol Phys. 1991;21(1):109–22. 10.1016/0360-3016(91)90171-y. 2032882 10.1016/0360-3016(91)90171-y

[19] Scarpa L, Buxbaum S, Kendler D, et al. The ^68^Ga/^177^Lu theragnostic concept in PSMA targeting of castration-resistant prostate cancer: correlation of SUVmax values and absorbed dose estimates. Eur J Nucl Med Mol Imaging. 2017;44(5):788–800. 10.1007/s00259-016-3609-9. 28083690 10.1007/s00259-016-3609-9

[20] Bodei L, Cremonesi M, Ferrari M et al. Long-term evaluation of renal toxicity after peptide receptor radionuclide therapy with 90Y-DOTATOC and 177Lu-DOTATATE: the role of associated risk factors [published correction appears in Eur J Nucl Med Mol Imaging. 2008;35(10):1928]. *Eur J Nucl Med Mol Imaging*. 2008;35(10):1847–56. 10.1007/s00259-008-0778-1 10.1007/s00259-008-0778-1 18427807

[21] Kuczynski EA, Sargent DJ, Grothey A, Kerbel RS. Drug rechallenge and treatment beyond progression-implications for drug resistance. Nat Rev Clin Oncol. 2013;10(10):571–87. 10.1038/nrclinonc.2013.158. 23999218 10.1038/nrclinonc.2013.158 PMC4540602

[22] Gafita A, Rauscher I, Retz M, et al. Early experience of Rechallenge 177Lu-PSMA Radioligand Therapy after an initial good response in patients with advanced prostate Cancer. J Nucl Med. 2019;60(5):644–8. 10.2967/jnumed.118.215715. 30442756 10.2967/jnumed.118.215715

[23] Violet J, Sandhu S, Iravani A, et al. Long-term follow-up and outcomes of Retreatment in an expanded 50-Patient single-center phase II prospective trial of ^177^Lu-PSMA-617 theranostics in metastatic castration-resistant prostate Cancer. J Nucl Med. 2020;61(6):857–65. 10.2967/jnumed.119.236414. 31732676 10.2967/jnumed.119.236414 PMC7262220

[24] Yordanova A, Linden P, Hauser S, et al. Outcome and safety of rechallenge [^177^Lu]Lu-PSMA-617 in patients with metastatic prostate cancer. Eur J Nucl Med Mol Imaging. 2019;46(5):1073–80. 10.1007/s00259-018-4222-x. 30474706 10.1007/s00259-018-4222-x

[25] Seifert R, Telli T, Lapa C, et al. Safety and Efficacy of Extended Therapy with [^177^Lu]Lu-PSMA: a German Multicenter Study. J Nucl Med. 2024;65(6):909–16. 10.2967/jnumed.123.267321. Published 2024 Jun 3. 38697669 10.2967/jnumed.123.267321

[26] World Medical Association. Declaration of Helsinki: ethical principles for medical research involving human subjects. JAMA. 2000;284(23):3043–5. 11122593

[27] Arzneimittelgesetz BGB. Nr. 185/1983, last revision BGBl. II Nr. 105/2015. https://www.ris.bka.gv.at/GeltendeFassung.wxe?Abfrage=Bundesnormen&Gesetzesnummer=10010441. Updated July 23, 2019.

[28] Österreichischer Verband für Strahlenschutz. Mitgliedsgesellschaft der International Radiation Protection Association. http://www.strahlenschutzverband.at/index.php?id=strahlenschutzrecht0. Updated July 23, 2019.

[29] Kraihammer M, Garnuszek P, Bauman A, et al. Improved quality control of [^177^Lu]Lu-PSMA I&T. EJNMMI Radiopharm Chem. 2023;8(1):7. 10.1186/s41181-023-00191-6. Published 2023 Mar 27. 36971890 10.1186/s41181-023-00191-6 PMC10043144

[30] European Pharmacopoeia. LUTETIUM (177Lu) ZADAVOTIDE GURAXETAN INJECTION. 06/2024:3170 (11th Edition).

[31] Common Terminology Criteria for Adverse Events. (CTCAE v5.0). https://ctep.cancer.gov/protocoldevelopment/electronic_applications/docs/CTCAE_v5_Quick_Reference_5x7.pdf (accessed on 14th April 2024).

[32] Scher HI, Morris MJ, Stadler WM, et al. Trial Design and objectives for castration-resistant prostate Cancer: updated recommendations from the prostate Cancer clinical trials Working Group 3. J Clin Oncol. 2016;34(12):1402–18. 10.1200/JCO.2015.64.2702. 26903579 10.1200/JCO.2015.64.2702 PMC4872347

[33] Wahl RL, Jacene H, Kasamon Y, Lodge MA. From RECIST to PERCIST: evolving considerations for PET response criteria in solid tumors. J Nucl Med. 2009;50(Suppl 1):S122–50. 10.2967/jnumed.108.057307. 10.2967/jnumed.108.057307 PMC2755245 19403881

[34] Seitz AK, Rauscher I, Haller B, et al. Preliminary results on response assessment using ^68^Ga-HBED-CC-PSMA PET/CT in patients with metastatic prostate cancer undergoing docetaxel chemotherapy. Eur J Nucl Med Mol Imaging. 2018;45(4):602–12. 10.1007/s00259-017-3887-x. 29185010 10.1007/s00259-017-3887-x

[35] Emmenegger U, Ko YJ. PSA-based treatment response criteria in castration-resistant prostate cancer: promises and limitations. Can Urol Assoc J. 2009;3(5):375–6. 10.5489/cuaj.1146. 19829728 10.5489/cuaj.1146 PMC2758513

[36] Gafita A, Heck MM, Rauscher I, et al. Early prostate-specific Antigen changes and clinical Outcome after 177Lu-PSMA Radionuclide Treatment in patients with metastatic castration-resistant prostate Cancer. J Nucl Med. 2020;61(10):1476–83. 10.2967/jnumed.119.240242. 32111687 10.2967/jnumed.119.240242

[37] Emmett L, John N, Pathmanandavel S et al. Patient outcomes following a response biomarker-guided approach to treatment using ^177^Lu-PSMA-I&T in men with metastatic castrate-resistant prostate cancer (Re-SPECT). *Ther Adv Med Oncol*. 2023;15:17588359231156392. Published 2023 Mar 1. 10.1177/17588359231156392 10.1177/17588359231156392 PMC9983078 36872949

[38] Giovanella L, Garo ML, Cuzzocrea M, Paone G, Herrmann K. Prognostic role of early prostate specific antigen changes after [^177^Lu]Lu-PSMA radioligand therapy of metastasized prostate cancer: a meta-analysis. Eur J Clin Invest. 2023;53(9):e14014. 10.1111/eci.14014. 37194605 10.1111/eci.14014

[39] Rahbar K, Boegemann M, Yordanova A, et al. PSMA targeted radioligand therapy in metastatic castration resistant prostate cancer after chemotherapy, abiraterone and/or enzalutamide. A retrospective analysis of overall survival. Eur J Nucl Med Mol Imaging. 2018;45:12–9. 29026946 10.1007/s00259-017-3848-4

[40] Bakht MK, Derecichei I, Li Y, et al. Neuroendocrine differentiation of prostate cancer leads to PSMA suppression. Endocr Relat Cancer. 2018;26(2):131–46. 10.1530/ERC-18-0226. Published 2018 Nov 23. 30400059 10.1530/ERC-18-0226

[41] Chen R, Wang Y, Zhu Y, Shi Y, Xu L, Huang G, Liu J. The added value of ^18^F-FDG PET/CT compared with ^68^Ga-PSMA PET/CT in patients with castration-resistant prostate Cancer. J Nucl Med. 2022;63(1):69–75. 10.2967/jnumed.120.262250. 34980667 10.2967/jnumed.120.262250 PMC8717199

[42] Weber M, Hadaschik B, Ferdinandus J, Rahbar K, Bögemann M, Herrmann K, Fendler WP, Kesch C. Prostate-specific Membrane Antigen-based imaging of castration-resistant prostate Cancer. Eur Urol Focus. 2021;7(2):279–87. Epub 2021 Jan 20. PMID: 33483289. 33483289 10.1016/j.euf.2021.01.002

[43] Patel GK, Chugh N, Tripathi M. Neuroendocrine differentiation of prostate Cancer-An Intriguing Example of Tumor Evolution at Play. Cancers (Basel). 2019;11(10):1405. 10.3390/cancers11101405. Published 2019 Sep 20. 31547070 10.3390/cancers11101405 PMC6826557

[44] Wang HT, Yao YH, Li BG, Tang Y, Chang JW, Zhang J. Neuroendocrine prostate Cancer (NEPC) progressing from conventional prostatic adenocarcinoma: factors associated with time to development of NEPC and survival from NEPC diagnosis-a systematic review and pooled analysis. J Clin Oncol. 2014;32(30):3383–90. 10.1200/JCO.2013.54.3553. 25225419 10.1200/JCO.2013.54.3553

[45] Jiborn T, Bjartell A, Abrahamsson PA. Neuroendocrine differentiation in prostatic carcinoma during hormonal treatment. Urology. 1998;51(4):585–9. 10.1016/s0090-4295(97)00684-5. 9586611 10.1016/s0090-4295(97)00684-5

[46] Deng X, Liu H, Huang J, et al. Ionizing radiation induces prostate cancer neuroendocrine differentiation through interplay of CREB and ATF2: implications for disease progression. Cancer Res. 2008;68(23):9663–70. 10.1158/0008-5472.CAN-08-2229. 19047143 10.1158/0008-5472.CAN-08-2229 PMC3100895

[47] Berruti A, Dogliotti L, Mosca A, et al. Circulating neuroendocrine markers in patients with prostate carcinoma. Cancer. 2000;88(11):2590–7. 10861438 10.1002/1097-0142(20000601)88:11<2590::aid-cncr23>3.0.co;2-d

[48] Mader N, Nguyen Ngoc C, Kirkgöze B, et al. Extended therapy with [^177^Lu]Lu-PSMA-617 in responding patients with high-volume metastatic castration-resistant prostate cancer. Eur J Nucl Med Mol Imaging. 2023;50(6):1811–21. 10.1007/s00259-023-06119-1. 36702927 10.1007/s00259-023-06119-1 PMC10119067

[49] von Eyben FE, Singh A, Zhang J, et al. ^177^Lu-PSMA radioligand therapy of predominant lymph node metastatic prostate cancer. Oncotarget. 2019;10(25):2451–61. 10.18632/oncotarget.26789. Published 2019 Mar 29. 31069008 10.18632/oncotarget.26789 PMC6497435

[50] Yadav MP, Ballal S, Sahoo RK, Tripathi M, Seth A, Bal C. Efficacy and safety of ^225^Ac-PSMA-617 targeted alpha therapy in metastatic castration-resistant prostate Cancer patients. Theranostics. 2020;10(20):9364–77. 10.7150/thno.48107. Published 2020 Jul 23. 32802197 10.7150/thno.48107 PMC7415797

