# Supplementary material for: Efficacy and safety of rechallenge with [177Lu]Lu-PSMA-I&T radioligand therapy in metastatic castration resistant prostate cancer
Source: Eur J Nucl Med Mol Imaging. 2024 Sep 3;52(1):354–65. doi: 10.1007/s00259-024-06905-5 (PMC11599357; doi:10.1007/s00259-024-06905-5)
Supplement: Supplementary file 1 — Supplementary Material 1 [file 259_2024_6905_MOESM1_ESM.docx]

**Supplementary File**


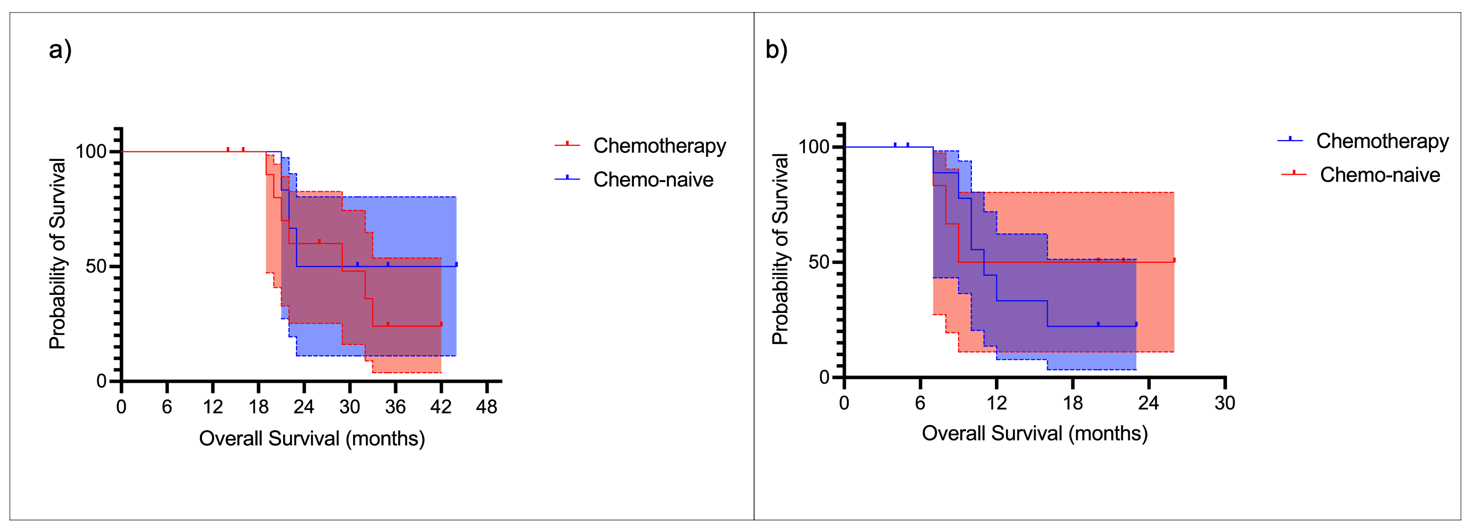


**Fig 1.** Kaplan-Meier curve comparisons showed no statistically significant differences in Overall Survival (OS) between patients who received chemotherapy prior to rechallenge and chemo-naive patients, whether a) cumulative OS from the start of initial treatment (median OS 29 vs. 33.5 months, p=0.62) or b) from the start of rechallenge (median OS 11 vs. 17.5 months, p=0.61).
